# Radial Increment of Beech (*Fagus sylvatica* L.) Is under a Strong Impact of Climate in the Continental Biogeographical Region of Croatia

**DOI:** 10.3390/plants12132427

**Published:** 2023-06-23

**Authors:** Tom Levanič, Damir Ugarković, Ivan Seletković, Mladen Ognjenović, Mia Marušić, Robert Bogdanić, Nenad Potočić

**Affiliations:** 1 Department for Forest Yield and Silviculture, Slovenian Forestry Institute, Večna pot 2, SI-1000 Ljubljana, Slovenia; 2 Faculty of Mathematics, Natural Sciences and Information Technologies, University of Primorska, Glagoljaška 8, SI-6000 Koper, Slovenia; 3 Faculty of Forestry and Wood Technology, Institute of Forest Ecology and Silviculture, University of Zagreb, Svetošimunska Cesta 23, HR-10002 Zagreb, Croatia; 4 Croatian Forest Research Institute, Division for Forest Ecology, Cvjetno naselje 41, HR-10450 Jastrebarsko, Croatia; 5 Department of BI, Analytics and Research, Njuškalo Ltd., Miroslava Miholića 2, HR-10000 Zagreb, Croatia

**Keywords:** climate change, tree growth, forest productivity, drought, European beech

## Abstract

European beech (*Fagus sylvatica* L.) is an important component of forests in the alpine and continental biogeographical regions of Croatia. This study aimed to (1) analyze the long-term response of beech to climate, (2) identify potentially critical climatic conditions that could negatively affect the radial increment (RI) and vitality of beech, and (3) evaluate differences in the response of beech between the two biogeographical regions in Croatia. We used the 16 × 16 km Croatian ICP Forests Level 1 network. On a total of 25 plots, we cored between 5 and 24 trees for dendrochronological analysis. Tree-ring widths (TRW) were measured and standardized using cubic spline. TRW chronologies for the two regions were calculated and correlated to the temperature and precipitation data and Standardized Precipitation and Evapotranspiration Index (SPEI) using bootstrapped correlations. Continental region precipitation from April to August and alpine region precipitation from June to August were significantly important for RI. Temperature was less important for RI than precipitation in both regions, but the importance of the negative impact of above-average temperatures in the continental region and the positive impact of above-average precipitation in the alpine region has increased over the last two decades. A comparison with the 3-month SPEI confirmed the significant influence of high temperatures and the lack of precipitation in August on the RI of beech trees in both regions.

## 1. Introduction

Meteorological and climatic conditions have direct and indirect effects on vegetation. The direct effects include responses to temperature, while the indirect effects primarily occur as soil-mediated phenomena, such as the influence of precipitation on soil moisture regimes [1,2,3,4]. Climate change likely represents the greatest threat to existing forest ecosystems in Europe [5]. However, there is considerable uncertainty regarding the magnitude and character of climate change, particularly at the regional level [6].

Extreme climatic events, such as droughts, are thought to be important in initiating changes in forest ecosystems [7]. Southeastern Europe is considered one of the most vulnerable hotspots, with an expected intensification of the severity and duration of droughts and heatwaves. This region has already experienced a high frequency of drought events. After the year 2000, significant droughts and heatwaves were observed in 2002, 2003, 2007–2008, 2011, and 2012 [8]. A trend of decreasing precipitation and increasing temperatures was already observed in Croatia during the 20th century and was further amplified in the beginning of the 21st century. In the decade from 2001 to 2010 alone, four drought events occurred [9], which is in agreement with the drying trend observed across the Mediterranean [10,11]. In the future, Croatia is expected to be hotter and drier, with considerable impacts anticipated for forest ecosystems [12,13]. Given that the effects of climate change on forests in Southern Europe are projected to be stronger and more rapid than in the rest of Europe, this area represents an ideal model for studying the impact of changing climatic conditions [14,15,16]. 

The quantification of forest ecosystem behavior in response to climate change is crucial for the maintenance, enhancement, and restoration of future forest ecosystem goods and services. Changes in forest composition resulting from anthropogenic and/or climatic impacts will undoubtedly affect future silviculture practices, forest management, and forest-based industry [17,18]. Therefore, it is important to determine the level of tree response to climate conditions. Radial increment is sensitive to environmental conditions and local and/or regional climate [19] and can therefore be used as an indicator of tree vitality [20].

In the changing environmental conditions of today, tree growth has become one of the most important parameters for assessing tree reaction and forest condition [21,22]. Similar to other indicators of vitality such as defoliation or foliar composition, tree increment is an integrative variable of tree response [23]. The growth of trees is a key ecological parameter of forests and thus of high importance as an indicator of forest condition [24]. 

Growth can be influenced by external as well as internal factors, making it a useful proxy for assessing the reaction of trees and stands to changes in site and environmental conditions [21]. Trees generally respond to environmental stresses by experiencing a decrease in increment, resulting from reduced photosynthetic activity due to limitations in the environment. This leads to altered carbon allocation. Studies on beech growth show that it is strongly limited by drought [25,26]. Etzold, et al. [27] documented reduced beech growth due to dry conditions in the period 2000–2005 in Switzerland.

Common beech is a dominant broadleaved tree species in both European and Croatian temperate forests. Although beech is adapted to various ecological conditions, it is considered to be a drought-sensitive species [28,29] and grows best in areas characterized by moderately warm summers and high levels of precipitation [30]. Climate change may even pose a threat to the survival of beech at the latitudinal and altitudinal limits of its distribution [30]. Some researchers hypothesize that the expansion of beech in Central Europe during the Holocene was triggered by a shift towards wetter and cooler climatic conditions [31]. 

Despite its ecological tolerance, the competitive capacity of beech may be affected by the increasing frequency and severity of hot, dry summers [28,32]. This impact is not limited to the edge of its distribution range but also extends to areas previously considered optimal for its growth [29,33]. On more extreme sites, such as those located between the Mediterranean and the Alps, summer drought generally has a negative effect on beech growth in the lowlands, while high-elevation sites experience a positive effect of summer temperatures on tree ring width [34]. Understanding the inter-annual growth patterns of beech can aid in predicting its future distribution under anticipated climate change scenarios [17,35].

Current predictions regarding the impacts of climate change on plant demography rely on the association between species’ current geographical distribution and corresponding climate characteristics. These predictions consider hypothetical constraints imposed by temperature and/or moisture availability extremes [36].

Dendroecological analyses typically utilize local data and provide valuable regional insights into growth responses to local habitat conditions and climate change. Successful upscaling of tree-ring data, however, requires dense networks covering the full range of bioclimatic and ecological conditions of the study species, as discussed by del Castillo et al. [37].

Based on these ideas, we hypothesize that both temperature and precipitation significantly influence the radial growth of common beech, with the actual (micro) climatic conditions of the specific area playing a crucial role and drought having the most detrimental effect. Our main objectives were as follows:

(1) to analyze the long-term growth response of beech to climate;
(2) to identify extreme drought events that have negatively impacted the radial increment of beech;
(3) to determine whether there are significant differences in the growth response of beech trees between the alpine and continental biogeographical regions of Croatia under changing climate conditions.

## 2. Results

### 2.1. Radial Increment in the Alpine and Continental Biogeographical Regions of Croatia

**Figure 1:**
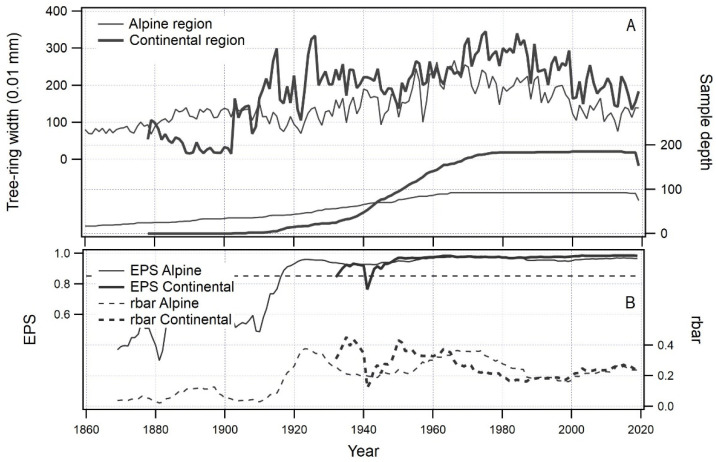
Tree-ring width chronologies for the alpine and continental biogeographical regions (panel (**A**)–left axis) with sample depth (panel (**A**)–right axis) and expressed population signal (EPS) (panel (**B**)–left axis) and average correlation between chronologies (rbar) (panel (**B**)–right axis). The dashed line in the lower panel represents the acceptance threshold of 0.85 for the EPS value. EPS values greater than or equal to 0.85 are sufficiently high to contain a meaningful common population signal for the studied region.

The results (Figure 1) show that radial increment is generally higher in the continental biogeographical region than in the alpine biogeographical region. The differences between the regions have remained stable over time, with radial increment mostly lower in the alpine region. However, there is a brief period between 1950 and 1970 when the two regions exhibit similar radial increments. Despite these differences, both tree-ring width chronologies show good agreement (t_BP_ = 5.49, GLK% = 68.30%) and display a common response overall, particularly during extreme years such as 2000, 2003, and 2013.

The expressed population signal [38] (EPS, Figure 1, lower panel) in both the alpine and continental biogeographical regions is high (>0.85) and stable from 1945 to 2020. This indicates that the climate-growth relationship calculation can be performed and that the results should have a meaningful statistical interpretation.

### 2.2. General Response of Radial Increment to Climate

Based on the high EPS value observed in the period 1945–2020 for both regions, we were able to perform a climate-growth analysis. The general pattern of the growth response of European beech to climate shows a smaller number of significant correlations between tree-ring widths and temperatures and precipitation for plots in the alpine region (Table 1). Correlations between tree-ring widths and temperatures and precipitation on the research plots in the continental region are significant, with higher absolute values compared to the alpine region (Table 1). The tree response pattern in both biogeographical regions is clear, indicating that precipitation has a positive influence on radial increment. In the alpine region, this influence is less pronounced than in the continental region, and the window where precipitation is significant is narrower compared to that in the continental region. In the alpine region, precipitation is important for radial growth during the summer months (June, July, and August), whereas in the continental region, this period extends from April to August. The correlations in the continental region also exhibit much higher absolute values compared to the alpine region (values shown in Table A1).

The influence of temperature on radial growth (Table 1) is negative in both biogeographical regions and less pronounced in the alpine region than in the continental region. In the alpine region, it is challenging to identify a clear pattern, as there are only indications that temperature has a generally negative influence on the radial growth of beech, while in the continental region, this negative influence is clearly present only in the summer months.

**Figure 2:**
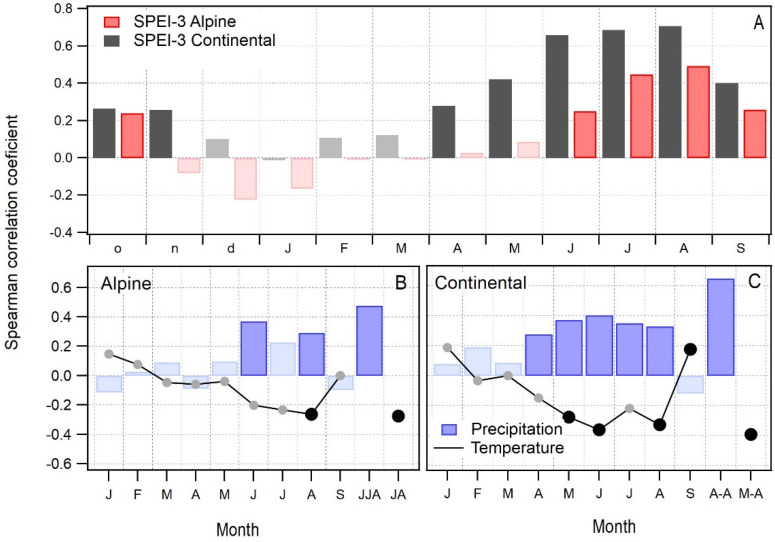
Bootstrapped correlation between radial increment and standardized precipitation and evapotranspiration index-SPEI (**A**) and average monthly temperature and monthly sum of precipitation in the alpine (**B**) and continental (**C**) biogeographical regions for the period 1950–2020. Correlations for the months January to September and different combinations of values are shown. Significant relationships (*p* < 0.05) are indicated by darker bars or emphasized black dots.

The response of radial increment to climate differs between the two studied regions. An analysis of the correlation between tree-ring widths and the 3-month Standardized Precipitation and Evapotranspiration index (SPEI-3, Figure 2A) reveals a significant correlation with the summer months, with August SPEI-3 showing the highest correlation regardless of the biogeographical region. In terms of absolute values, the correlations in the continental region (r = 0.71) are higher than in the alpine region (r = 0.49). This indicates a strong dependency between radial increment and climatic conditions and indicates that above-average summer temperatures and a lack of precipitation, in combination with increased evapotranspiration, reduce the radial growth of beech in both biogeographical regions.

In the alpine region (Figure 2B), correlations between tree-ring widths and average monthly temperature, monthly sum of precipitation, and their combinations are lower compared to the continental region. In the continental region (Figure 2C), precipitation between April and August is the most important factor for radial increment (r = 0.65), meaning that more precipitation in that period results in a larger radial increment. A similar influence of precipitation was observed in the alpine region, but here precipitation within a shorter timespan (June to August, r = 0.48) was most important for radial growth.

Regardless of the biogeographical region, the response of radial increment to above-average temperatures is generally negative. In the continental region, temperatures are important, but less so than precipitation (Figure 2C). We found significant correlations between radial increment and temperatures in the summer months (May, r = −0.28; June, r = −0.36; and August, r = −0.33) and for the period from May to August (r = −0.39).

In the alpine region, temperatures are only important when considered as a combination of July and August temperatures (r = −0.28) or as August temperature alone (r = −0.26).

### 2.3. Temporal Stability of the Climate Signal and Spatial Outreach of the Climate Signal in Beech Tree Rings

Unlike the general response to climate (Figure 2), which illustrates how beech radial increment responds to climate parameters over a longer time span, moving window statistics provide insights into the temporal stability of the response and whether certain climate factors have gained importance as we approach the present time (e.g., the influence of a changing climate).

**Figure 3:**
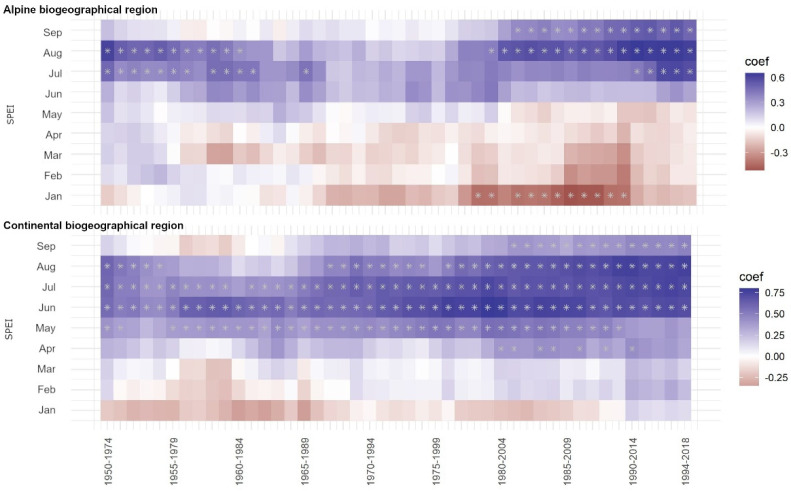
Temporal stability of bootstrapped correlations between SPEI and radial increment in the alpine (**top**) and continental (**bottom**) biogeographical regions for the period 1950–2018. Squares in red indicate a negative correlation, and squares in blue indicate positive correlations. Squares with stars denote a statistically significant (95% confidence) correlation. Each time window represents 25 years, with a 1-year lag (for clarity, only every 5th time window is displayed).

In the continental region, moving window correlation statistics (Figure 3, bottom) reveal a high, positive, significant, and stable relationship between radial increment and SPEI in June and July throughout the entire studied period. This indicates that above-average precipitation, in combination with average temperature and evapotranspiration, positively impacts beech growth in the continental biogeographical region. Moreover, this influence has become increasingly important over time, as indicated by higher absolute values of the moving correlations as we approach the present. In contrast, the alpine biogeographical region shows a different response than the continental region. The moving window correlation shows a significant response only in July and August, but the response is not stable over time. Specifically, the period between 1960 and 2004 lacks any significant response during these months. However, it is worth noting that the importance of August SPEI has been increasing as we approach the present, indicating that precipitation (in combination with temperature and evapotranspiration) is gaining importance and will likely play an important role for the growth of beech in the future.

**Figure A1:**
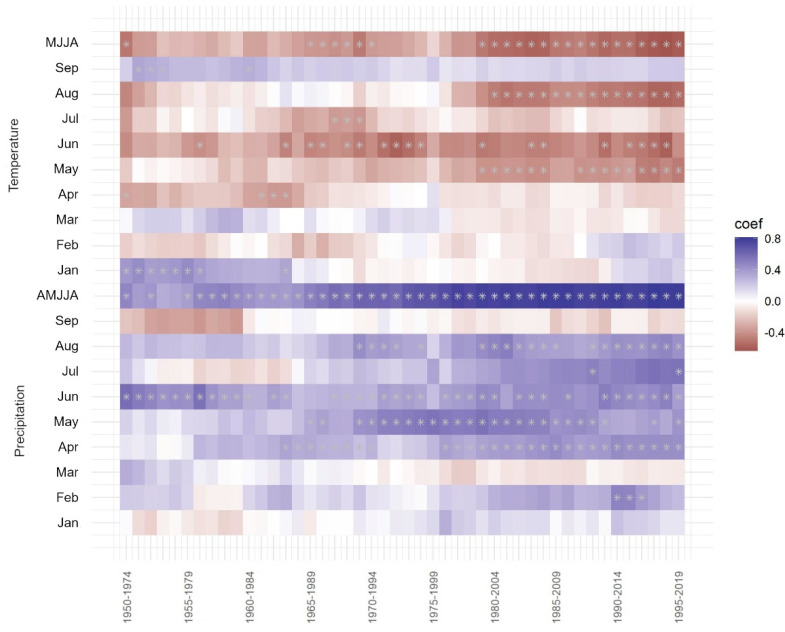
Temporal stability of bootstrapped correlations of temperature and precipitation with radial increment in the continental biogeographical region. Squares in red show negative correlations, and squares in blue show positive correlations. Squares with stars indicate statistically significant (95% confidence) correlations. Every time window represents 25 years, with a 1-year lag (for the sake of clarity, only every 5th time window label is displayed on *x*-axis).

The relationship between radial increment and April to August precipitation in the continental region was found to be high, significant, and stable throughout the entire studied period (Figure A1). Conversely, temperatures between May and August have only become important in recent decades, indicating that the influence of above-average temperatures in the continental region is gaining importance and may pose a serious threat to the growth of beech in the future.

**Figure A2:**
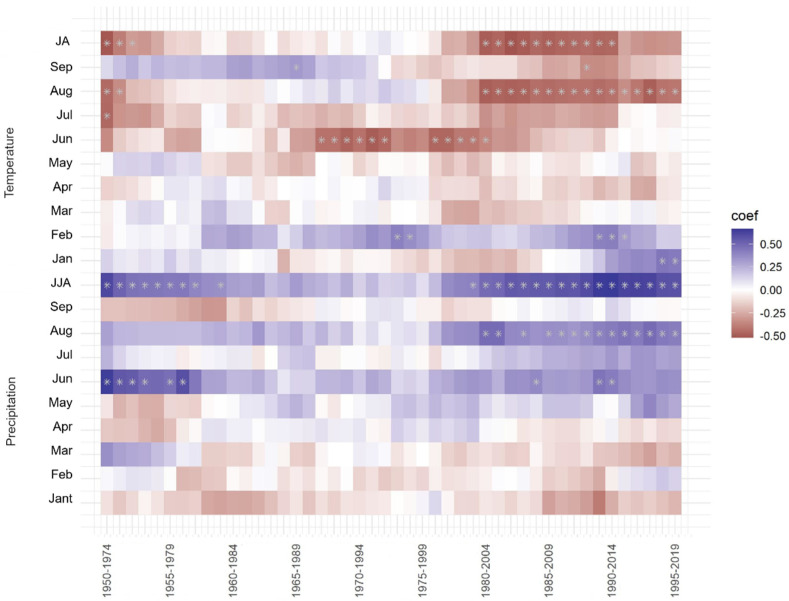
Temporal stability of bootstrapped correlations of temperature and precipitation with radial increment in the alpine biogeographical region. Squares in red show negative correlations, and squares in blue show positive correlations. Squares with stars indicate statistically significant (95% confidence) correlations. Every time window represents 25 years, with a 1-year lag (for the sake of clarity, only every 5th time window label is displayed on *x*-axis).

In the alpine region, the radial increment of beech has been affected by precipitation only in recent decades, although this influence is not as pronounced as in the continental region. In addition, the critical months for radial growth (June to August) differ from those in the continental region (April to August, Figure A2). In recent decades, temperatures have also been gaining importance for radial growth in the alpine region.

**Figure 4:**
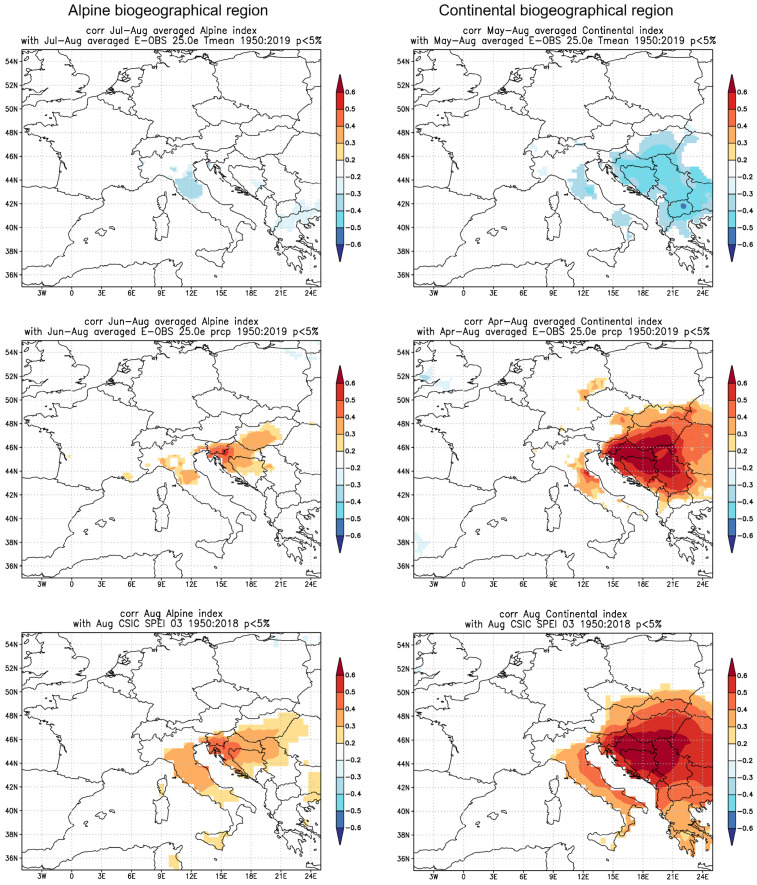
Spatial correlations between radial increment and the E-OBS meteorological dataset for average monthly temperature, monthly sum of precipitation for the period 1950–2019 and SPEI index for the period 1950–2018 for the alpine (**left** panel) and continental (**right** panel) biogeographical regions. The top row represents temperature, the middle row represents precipitation, and the bottom row represents SPEI. Only significant combinations are presented, which include the combination of July–August average monthly temperature and May–August monthly sum of precipitation for the alpine biogeographical region, and the combination of June–August average monthly temperature and April–August monthly sum of precipitation for the continental biogeographical region. In both regions, the analysis includes August SPEI.

We also performed a spatial analysis of the climate-growth relationship by correlating grid cells of meteorological data and indexed tree-ring width series for the period 1950–2019 for temperature and precipitation, and from 1950 to 2018 for SPEI (Figure 4). The spatial extent of the climate signal in tree-rings is smaller for the alpine region (Figure 4, left panel) than for the continental region (Figure 4, right panel), both in terms of spatial extent and the magnitude of the correlation values. In particular, the temperature signal in the alpine region is spatially weak and inconclusive. The precipitation signal in the alpine region has a slightly larger spatial extent and corresponds well with the Dinaric Mountain range. The spatial extent of the tree-ring width correlation with SPEI is the largest of all three analyzed parameters in the alpine biogeographical region, although in terms of absolute values it is not significantly higher.

In contrast, the spatial extent of the climatic signal in beech tree rings is extensive in the continental region (Figure 4, right panel), particularly for precipitation, covering a large area of Pannonian lowland, including the neighboring countries of Hungary, Bosnia and Herzegovina, and Serbia. The signal corresponds well with the precipitation pattern in the Pannonian lowland, indicating that the lowland and the hills surrounding it are under significant threat from summer drought. The spatial extent of correlation between the tree-ring width and SPEI is the highest among the three studied parameters.

### 2.4. Response of the Radial Increment of Beech in Extreme Years

**Figure 5:**
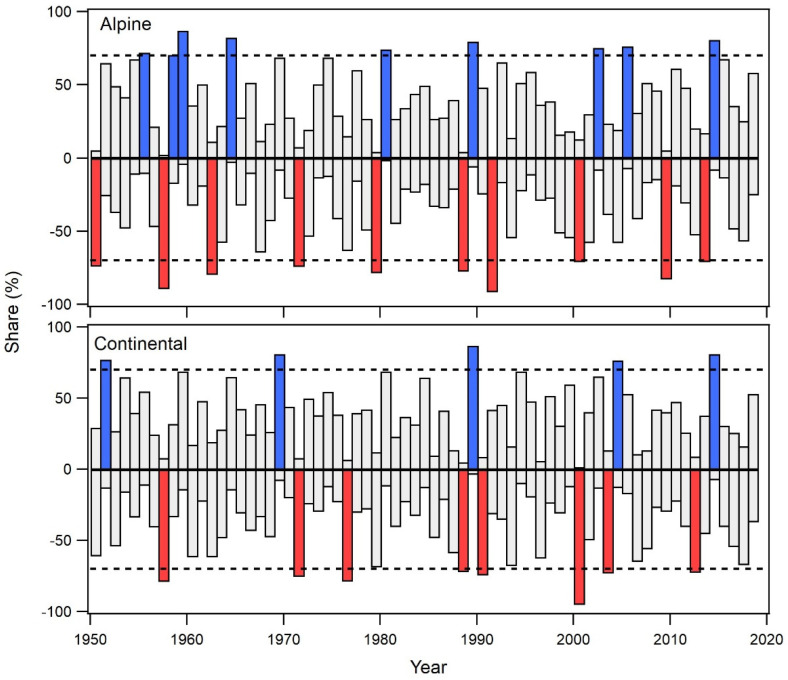
Pointer years in the alpine and continental biogeographical regions (red bars indicate negative pointer years, blue bars indicate positive pointer years, and grey bars indicate non-significant responses). The upper and lower horizontal dashed lines represent the 70% threshold value needed for a pointer year to be considered significant.

In our study area, all beech trees on all sites show a synchronous response to climate conditions. By means of pointer year analysis (PY), where we identify plot-wise common narrow or wide tree rings, we identified several positive and negative pointer years in both the alpine and continental biogeographical regions (Figure 5). In general, the alpine region has a greater number of PY (9 positive and 10 negative) than the continental region. The continental region has five positive and eight negative PY, which is unexpected given the weak climate signal in the tree-ring width chronology of beech in the alpine region.

Only a few PY were common to both biogeographical regions. The common positive PY were 1989 and 2014, while the common negative PY included 1957, 1971, 1988, and 2000.

In the alpine region, negative PY are associated with below-average precipitation and above-average temperature, while positive PY are linked to wet to extremely wet conditions and average to below-average temperatures in the growing season months (Table 2, alpine region). A similar response of beech is also observed in the continental region (Table 2, continental region). Years identified as negative PY are characterized by dry to very dry spring and summer months and above-average temperatures in that period, while positive PY are always associated with at least average precipitation and average to below-average temperatures.

## 3. Discussion

Dendroecological methods provide valuable insights into the response of forest trees, as tree-ring widths are strongly controlled by climatic events and as such are an important indicator of changes in the environment and climate [40].

In terms of growth synchronicity, the radial growth of beech in the alpine and continental biogeographical regions is comparable. However, there are differences in terms of absolute radial increment. This discrepancy could be attributed to the fact that the studied beech trees in the alpine region originate from older forest stands where the forest stand structure is closer to old-growth forests [30]. In contrast, in the continental region, beech forest management is more intense, and forest stands are mostly even aged and composed of younger trees, which usually have a more pronounced response to climate than older trees [41]. Both biogeographical regions exhibit an increase in radial growth from the 1960s to the 1980s. However, a decrease in radial growth is observed after 1980, which is consistent with studies of beech radial growth in other parts of Europe [32,37]. Model predictions show that beech growth is expected to decline by as much as 40–50% in the Balkan Peninsula and in the Apennines in the period 2040–2070 [37]. Changing climatic conditions, characterized by more frequent hot spells and drought periods, are among the most important factors contributing to the decline in radial growth [32,42].

When we merge the information gathered with the pointer year analysis and the results from the climate-growth relationship (including temporal analysis using moving window statistics), we can conclude that beech exhibits a strong general climate signal in tree rings and is highly responsive to extreme climatic events, such as extremely dry and hot years.

Beech is a distinct mesophyte, which means that it is not adapted to either particularly dry or particularly wet environments and can therefore benefit from large amounts of precipitation in the summer months [30]. However, the predicted increase in drought events, particularly in the continental biogeographical region, could have a significant negative impact on beech growth and regeneration [32]. Our findings are in accordance with research conducted in Hungary, where the availability of water (precipitation) in the May–July period is critical for the survival of beech [43]. Similar patterns have been observed in Italy, where precipitation plays a major role along the Apennine Mountains [44,45]. In the alpine biogeographical region, temperature appears to be of marginal importance compared to the continental region, where it plays a more important role, especially when combined with a lack of precipitation, as indicated by greater values of the correlations with SPEI-3.

Trees are always good indicators of climatic and environmental conditions. Tree growth in extremely dry and wet years not only shows their immediate response to the unfavorable conditions but also the conditions limiting the survival of the species, since tree vitality can be defined as the ability of a tree to assimilate, survive stress, and react to changing conditions [46]. Therefore, indicators of tree life, growth, and development have to be used [24,47]. Beech growth is above average in years with more precipitation during the growing season and below average in years with low amounts of precipitation and above-average temperatures. Based on the response of beech in PY, it is evident that the change of climate towards warmer and dryer conditions predicted for the future [48] will have dramatic consequences on European beech growth, and it is entirely possible that beech will not be able to adapt to the changed climatic conditions and that we will face increased mortality of beech trees, especially in the continental biogeographical region. Climate change and extreme climatic conditions such as drought are predicted to lead to a decrease in suitable forest sites for European beech [49]. It is predicted that 56–96% of today’s beech forests could be out of their current bioclimatic niche by 2050 [43], mostly at the southern border of common beech’s range [50]. Extreme climatic events, such as heat waves and dry periods, are expected to occur with increased frequency in Croatia as a whole and even more frequently in the continental biogeographical region of Croatia [51]. Severe and recurrent droughts have been identified as a major factor contributing to accelerated rates of tree decline and forest mortality in Europe [52].

Compared to the continental biogeographical region, the alpine biogeographical region is less exposed to increasing temperatures and a lack of precipitation in the summer months. According to the regression models [53,54], precipitation amounts are lower at lower altitudes, and air temperature is higher in the continental region. This observation is in line with the fact that the climate-growth response of beech in the alpine biogeographical region is less obvious, as correlations between tree-ring widths and different meteorological parameters were not as high as in the continental region. With regards to the effect of droughts, a higher number of dry years is recorded at meteorological stations at lower altitudes in comparison with higher altitudes [55]. This indicates that growing conditions for beech in the alpine region are more favorable than in the continental region, which is clearly visible in the climate-growth response statistics. Similar results were obtained by Kolář et al. [56] for the eastern part of Czech Republic, where beech sites above 800 m are more temperature dependent and sites below 800 m more precipitation dependent [56].

To summarize, the radial growth of beech trees in Croatia’s alpine and continental biogeographical regions is affected by climate change, especially in the continental region. Climate influences radial increment to such a degree that further intensification of climate warming may seriously impact the future productivity of European beech in the continental region of Croatia.

## 4. Materials and Methods

### 4.1. Research Plots

**Figure 6:**
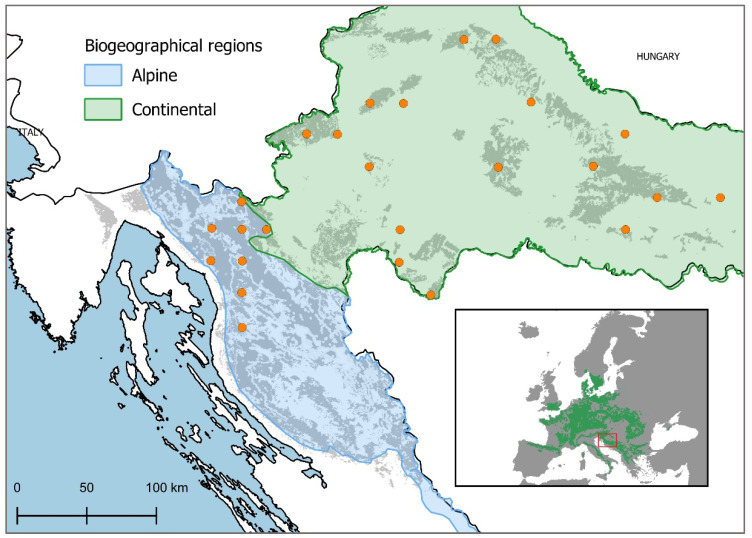
Locations of research plots within the ICP Forests Level I plot grid. The sampling locations chosen for the tree-ring width analysis are represented by orange circles. The blue area indicates the plots in the alpine biogeographical region, while the green area represents the plots in the continental biogeographical region. The smaller map (lower right corner) represents the location of Croatia in the wider geographical context.

We used the infrastructure of UNECE ICP Forests in Croatia for our field work. The 16 × 16 km ICP Forests grid has a pure systematic sampling design, which extends across Europe (referred to as Level I plots). The plots do not have a fixed area: 24 trees are chosen for assessment using a cross-cluster system [57]. Besides defoliation, this database contains information on plot characteristics (location, topography, stand age). For the tree-ring width study, a set of 25 plots, 8 in the alpine and 17 in the continental biogeographical regions of Croatia, were chosen based on the selection criteria that at least 5 out of the 24 trees were European beech (*Fagus sylvatica* L.) (Figure 6). Basic information about the sampling locations is presented in Table A2.

### 4.2. Sample Collection and Tree-Ring Width Analysis

All European beech trees on the selected Croatian Level I plots (Figure 6) were cored (two cores per tree) following methods described by Stokes and Smiley [58].

Each core was air-dried, mounted, and sanded with progressively finer sanding paper (up to 600 grit) on an industrial belt sander to achieve a highly polished surface and excellent visibility of tree rings [58]. The cores were then digitized using an ATRICS system [59], and the annual radial growth was measured to the nearest 0.01 mm using CDendro and CooRecorder software v. 9.8.1 (Cybis, Stockholm, Sweden). Each tree-ring series underwent visual and statistical crosschecking using PAST-5 software v. 5.0.610 (SCIEM, Vienna, Austria). In case of any dating or synchronization errors, appropriate corrections were made in CDendro software, and measurements were repeated if necessary.

Individual tree-ring width series were standardized to remove long-term trends [60,61] using a 67% cubic smoothing spline with a 50% frequency cut-off in the dplR library [62] of the R program [63]. Departures of the measured values from the regression curve were calculated as the quotient between the measured tree-ring width and fitted value, resulting in a dimensionless index with a mean of 1. By doing this, we eliminated factors that were not related to climate, such as tree age and the effects of stand dynamics [64]. Index values were pre-whitened using an autoregressive model that was selected based on the minimum Akaike criterion and were combined across all series using a biweight robust estimation of the mean in order to exclude the influence of outliers. The dplR produces two types of chronologies: standardized (STD) and residual (RES). In this research, we used the RES chronology, which is a robust estimate of the arithmetic mean with autocorrelation mathematically removed [65].

Composite chronologies for the alpine and continental biogeographical regions were compiled as follows: (1) all sites from one biogeographical region were merged into a single file and visually and statistically synchronized and cross-dated; (2) they were then standardized using the above-described procedure in the dplR library of the R program; and finally, (3) three different chronologies for each biogeographical region were produced: a raw data chronology, a standardized chronology, and a residual chronology.

### 4.3. Meteorological Data

Climatic data were collected from the KNMI Climate Explorer website [66,67]. For temperature and precipitation, we used monthly gridded data (0.25 × 0.25° grid) from the E-OBS database [68] covering the period between 1920 and the present. For the drought index, we chose the Standardized Precipitation and Evapotranspiration Index (SPEI) for the period 1901–2018 from the CSIC [69,70], also available at KNMI Climate Explorer.

To identify potential anomalies between gridded and local climate data, we compared the gridded meteorological climate data with shorter local climate data. We used three meteorological stations located in the alpine region (Delnice, Gospić, and Ogulin) and three meteorological stations in the continental region (Zagreb, Bjelovar, and Požega) for comparison with the gridded data. To verify positive and negative pointer years in tree rings, an analysis of monthly and annual climate anomalies (precipitation and temperature percentiles) was conducted according to the modified Conrad–Chapman method [71] based on the deviation from the multi-year average of the period 1961–1990.

**Figure 7:**
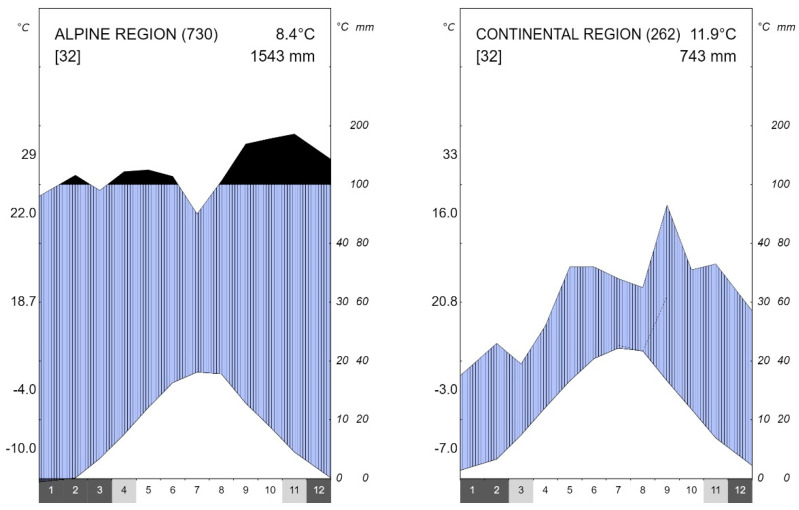
Climate diagrams for the alpine and continental biogeographical regions in Croatia for the period 1990–2021. Black color within figure—months with precipitation above 100 mm; blue color—wet, humid period. Dark grey color on *x*-axis—months with mean minimum air temperature <0 °C; light grey color—months with absolute minimum air temperature <0 °C. Numbers on the left *y*-axis from top to bottom: absolute maximum air temperature, mean maximum air temperature, average fluctuation of air temperature, mean minimum air temperature, and absolute minimum air temperature.

The alpine region has a maritime precipitation regime where less than 50% of precipitation falls in the warm part of the year. Precipitation has two maxima, one in early spring and the other in late autumn. It is the area with the highest values of precipitation in Croatia [30]. The continental region in Croatia, on the other hand, has a continental precipitation regime where more than 50% of the precipitation falls in the warm part of the year. Precipitation maxima occur in spring and late summer (Figure 7).

### 4.4. Statistical Data Processing

The expressed population signal (EPS) was used to assess the representation of a small sample in relation to the signal of the entire population [38]. EPS values range between 0 and 1, with values greater or equal to 0.85 considered high enough to indicate a common signal across the population [38]. This common signal can be related to environmental or climatological factors; however, in many cases, it is a climate signal contained within tree rings (see Section 2.2). In this study, EPS was used as a measure of the common signal in the composite chronology.

After de-trending, removing autocorrelation, and calculating EPS, tree-ring index chronologies were compared to average monthly temperatures, monthly sum of precipitation, and 3- and 6-month SPEI using a bootstrapped correlation calculation in the treeclim library [72] of the R program [63]. Additionally, several combinations of monthly temperature and precipitation data were tested against the standardized tree-ring widths to identify the best possible combination of influential climate variables. A 25-year window with a 1-year overlap for the calculation of the bootstrapped correlation between monthly temperature, precipitation, SPEI, and tree-ring widths was used to assess the temporal stability of the climate-growth relationship. Two SPEI indices, on a 3- and 6-month scale, were tested, and after careful examination of results, it was decided to use only the 3-month SPEI for further analysis.

Spatial analysis based on the correlation between standardized tree-ring widths and the gridded meteorological data (average monthly temperature, monthly sum of precipitation, and 3-month SPEI) (source of data: KNMI Climate Explorer) was used to test the spatial strength (outreach) of the climate signal in tree rings. Single months and combinations of months were used in the spatial analysis. Each grid cell statistic was tested for significance, and where correlations were statistically significant (*p* < 0.05), the value was displayed on the map [66].

Pointer year analysis, which examines extreme events in tree-ring width chronologies, was performed for chronologies from both the alpine and continental biogeographical regions. We used a slightly modified criteria for pointer year selection, as described in Schweingruber et al. [73]. A year was recognized as a pointer year when 70% of at least 70 trees per biogeographical region responded with an increase or decrease in tree-ring width in comparison to the prior year. The dplR library in R was used for the calculation of the pointer years.

Statistical analysis was conducted using the R libraries dplR [62] and treeclim [72], and graphs were created using the IgorPRO program. Climate diagrams, following Walther [74], were created in the the KlimaSoft 1.0.6 program (github.com/mfrntic/klimasoft, accessed on 13 June 2023). Descriptive statistics of climate data were performed using the Statistica 13.4.0 program (TIBCO Software Inc, Palo Alto, CA, USA).

## Figures and Tables

**Table 1:** General pattern of climate-growth responses for each research plot for the period 1950–2020. Precipitation is indicated by the blue color, and temperatures are indicated by the light red color. The sign indicates the direction of the correlation, with “+” representing a positive correlation and “−” representing a negative correlation. Months are denoted by single capital letters from J (January) to S (September).

Alpine Biogeographical Region																		
Plot no.	Precipitation									Temperature								
	J	F	M	A	M	J	J	A	S	J	F	M	A	M	J	J	A	S
20					+							−		−				
21										−								
22						+		+										
23													−					
24						+		+										
25							+	+			−							
26						+		+							−		−	
31								+										
**Continental biogeographical region**																		
38		+			+	+	+	+										
47					+		+	+						−				
59					+	+	+	+										
60		+			+	+	+	+									−	
69		+		+	+	+	+	+						−	−		−	
71	No response detected																	
72						+									−			
80		+		+	+										−			
88		+		+	+										−			
94						+								−	−			
97		+			+			+						−	−		−	+
106				+	+	+	+	+						−	−		−	
120						+									−	−		
125				+	+									−		−		
128		+						+							−		−	
131				+		+		+		−				−				
139				+		+		+		−				−	−	−	−	+

**Table 2:** Temperature (above) and precipitation (below) percentiles for pointer years in the period 1980–2019. Lower percentiles indicate drier or warmer months or years. The colors of the classification correspond to the internationally accepted color scale for extreme climate conditions [39].

	Years	Alpine region–months									Year		Percentiles	Classification
		I	II	III	IV	V	VI	VII	VIII	IX			˂2	extremely cold
Negative	1988	98	69	42	52	67	29	98	86	51	85		2–9	very cold
pointer years	1991	68	19	94	20	1	74	98	88	92	55		9–25	cold
	2000	39	81	80	99	97	100	55	100	76	100		25–75	normal
	2009	38	51	65	98	99	83	97	99	88	99		75–91	warm
	2013	75	33	22	90	51	87	99	98	55	96		91–98	very warm
Positive	1989	80	88	95	83	39	9	63	62	51	93		>98	extremely warm
pointer years	2014	98	91	95	97	58	92	75	73	54	100			
		Continental region–months												
		I	II	III	IV	V	VI	VII	VIII	IX	Year			
Negative	1988	97	76	43	31	61	32	96	88	56	77			
pointer years	1990	65	96	94	41	80	44	41	79	19	95			
	2000	42	83	71	99	87	97	58	100	59	100			
	2003	42	7	68	53	100	100	99	100	51	99			
	2012	88	7	94	87	83	100	100	100	95	100			
Positive	1989	55	82	93	84	27	5	76	65	46	88			
pointer years	2014	98	88	96	93	49	91	88	71	57	100			
	Years	Alpine region–months									Year		Percentiles	Classification
		I	II	III	IV	V	VI	VII	VIII	IX			˂2	extremely dry
Negative	1988	52	96	49	14	45	66	4	70	48	12		2–9	very dry
pointer years	1991	34	46	9	25	99	31	50	8	27	33		9–25	dry
	2000	5	13	44	7	12	4	93	3	49	15		25–75	normal
	2009	71	73	91	67	7	47	36	13	13	46		75–91	wet
	2013	98	98	99	7	91	6	5	27	86	90		91–98	very wet
Positive	1989	3	82	52	95	98	86	85	99	37	100		>98	extremely wet
pointer years	2014	98	100	16	96	65	55	97	95	89	100			
		Continental region–months												
		I	II	III	IV	V	VI	VII	VIII	IX	Year			
Negative	1988	50	96	64	34	13	9	12	51	57	13			
pointer years	1990	24	24	29	42	13	57	15	13	94	22			
	2000	8	21	19	15	28	4	42	8	53	3			
	2003	54	18	1	4	5	4	34	18	57	1			
	2012	14	24	1	17	89	35	37	6	71	21			
Positive	1989	7	31	40	51	93	46	51	95	41	29			
pointer years	2014	46	100	3	84	83	50	80	97	100	100			

## Data Availability

The data presented in this study are, due to legal reasons, only partly available on request from the corresponding author.

## Appendix A

**Table A1:** Correlations between tree-ring width and average monthly temperature, monthly sum of precipitation, and 3-month SPEI for the alpine (A) and continental (C) biogeographical regions.

		Precipitation								
Plot no.		Jan	Feb	Mar	Apr	May	Jun	Jul	Aug	Sep
20	A	−0.09	−0.14	0.02	0.09	−0.17	0.12	0.07	−0.07	−0.02
21	A	−0.03	0.03	0.15	−0.09	−0.13	0.20	0.19	0.11	0.05
22	A	−0.19	−0.13	0.02	0.00	0.17	0.29	0.21	0.28	−0.30
23	A	−0.10	−0.22	0.13	0.11	−0.07	0.28	0.11	0.03	−0.19
24	A	0.03	0.12	0.19	−0.12	0.07	0.33	0.17	0.20	−0.18
25	A	−0.14	−0.04	0.00	−0.01	0.01	0.21	0.32	0.25	−0.11
26	A	−0.09	−0.02	0.03	0.00	0.05	0.34	0.10	0.33	−0.01
31	A	−0.05	0.05	−0.08	0.06	0.20	0.13	0.19	0.25	−0.01
38	C	−0.06	0.22	0.08	0.20	0.47	0.48	0.39	0.37	−0.19
47	C	−0.15	0.09	−0.16	0.16	0.27	0.12	0.27	0.20	−0.24
59	C	−0.12	0.14	0.08	−0.04	0.32	0.44	0.22	0.36	−0.24
60	C	0.02	0.23	−0.08	0.21	0.46	0.27	0.30	0.61	−0.03
69	C	0.02	0.29	0.24	0.18	0.48	0.37	0.49	0.43	−0.13
71	C	−0.09	0.07	0.10	0.10	0.25	0.30	0.30	0.22	−0.18
72	C	−0.07	0.16	0.19	0.20	0.31	0.20	0.28	0.19	−0.14
80	C	0.08	0.33	0.17	0.14	0.33	0.33	0.24	0.16	0.08
88	C	0.13	0.09	0.28	0.25	0.32	0.47	0.23	0.29	−0.04
94	C	0.08	0.18	0.05	0.10	0.27	0.23	0.06	0.20	0.12
97	C	0.07	0.26	0.00	0.19	0.15	0.02	0.16	0.28	−0.08
106	C	0.02	0.08	0.14	0.30	0.38	0.43	0.29	0.27	−0.10
120	C	0.07	0.17	0.06	0.00	0.21	0.42	0.05	0.08	−0.09
125	C	0.05	0.19	−0.07	0.19	0.34	0.20	0.01	0.32	0.03
128	C	0.02	0.26	−0.08	0.14	0.25	0.20	0.18	0.46	−0.15
131	C	−0.07	0.10	−0.06	0.19	0.29	0.38	0.15	0.25	−0.24
139	C	−0.01	0.15	−0.08	0.27	0.19	0.35	0.20	0.27	−0.15
		Temperature								
Plot no.		Jan	Feb	Mar	Apr	May	Jun	Jul	Aug	Sep
20	A	0.12	0.17	0.19	−0.06	0.26	−0.07	−0.09	0.08	−0.14
21	A	0.33	0.05	0.02	0.00	0.14	−0.09	−0.15	−0.07	0.06
22	A	0.01	0.01	0.06	0.02	−0.19	−0.19	−0.24	−0.34	0.07
23	A	−0.01	−0.10	−0.01	−0.22	0.02	−0.19	−0.25	−0.11	0.08
24	A	0.15	0.10	−0.07	0.05	−0.04	−0.18	−0.15	−0.19	0.04
25	A	0.10	0.13	−0.06	−0.12	0.01	−0.14	−0.20	−0.21	−0.10
26	A	0.01	0.05	−0.07	−0.20	0.01	−0.24	−0.22	−0.30	−0.14
31	A	−0.02	−0.01	−0.05	0.08	−0.04	−0.05	−0.10	−0.08	0.00
38	C	0.18	0.04	0.09	0.04	−0.13	−0.31	−0.12	−0.20	0.16
47	C	0.10	0.16	0.06	0.00	−0.08	−0.13	0.07	−0.24	0.18
59	C	0.16	−0.05	0.06	−0.02	−0.05	−0.18	−0.14	−0.27	0.16
60	C	0.23	−0.01	0.00	0.05	−0.22	−0.27	−0.11	−0.35	0.03
69	C	0.28	0.11	−0.05	0.02	−0.29	−0.39	−0.20	−0.33	0.09
71	C	0.19	−0.06	−0.12	0.02	−0.16	−0.27	−0.21	−0.20	0.22
72	C	0.02	−0.19	0.06	0.02	−0.24	−0.25	−0.16	−0.13	0.20
80	C	0.26	−0.01	0.13	−0.05	−0.16	−0.29	−0.21	−0.24	0.09
88	C	0.20	0.11	0.10	−0.01	−0.13	−0.31	−0.23	−0.23	0.09
94	C	0.11	0.19	0.04	0.08	−0.22	−0.25	−0.02	−0.09	0.13
97	C	0.05	−0.18	−0.11	−0.03	−0.28	−0.26	−0.10	−0.22	0.13
106	C	0.03	0.00	0.00	−0.22	−0.28	−0.35	−0.22	−0.34	0.20
120	C	0.13	0.04	0.08	−0.20	−0.13	−0.25	−0.18	−0.21	0.21
125	C	0.16	0.03	0.09	−0.22	−0.21	−0.23	−0.10	−0.24	−0.06
128	C	0.05	0.01	0.08	−0.09	−0.17	−0.29	−0.21	−0.32	0.07
131	C	−0.07	−0.09	−0.10	−0.31	−0.15	−0.29	−0.22	−0.27	0.09
139	C	−0.15	−0.13	−0.01	−0.16	−0.23	−0.35	−0.33	−0.30	0.23
		SPEI−3								
Plot no.		Jan	Feb	Mar	Apr	May	Jun	Jul	Aug	Sep
20	A	−0.07	−0.03	−0.06	−0.02	−0.01	−0.04	−0.03	0.01	0.06
21	A	−0.25	−0.30	−0.17	−0.14	−0.08	−0.09	−0.07	−0.03	−0.02
22	A	0.16	−0.19	−0.24	−0.13	−0.12	−0.07	−0.03	0.07	0.06
23	A	0.19	−0.05	−0.13	0.00	0.02	0.03	−0.03	−0.01	−0.01
24	A	0.10	−0.03	−0.06	−0.01	0.00	0.07	0.08	0.14	0.10
25	A	0.00	−0.06	−0.10	−0.08	−0.10	−0.05	−0.04	−0.03	−0.05
26	A	−0.03	−0.06	−0.03	−0.01	−0.01	0.00	−0.11	0.11	−0.04
31	A	0.12	0.02	−0.01	0.12	0.23	0.35	0.27	0.16	−0.03
38	C	0.15	−0.05	−0.05	0.06	0.08	0.11	−0.09	−0.06	−0.16
47	C	0.02	−0.27	−0.29	−0.19	−0.14	−0.06	−0.03	0.02	0.01
59	C	0.19	−0.09	−0.06	−0.13	−0.06	−0.04	−0.09	−0.07	−0.12
60	C	0.11	−0.15	−0.12	−0.10	−0.12	−0.04	−0.06	0.02	−0.09
69	C	−0.07	−0.08	−0.10	−0.04	−0.06	0.00	−0.05	0.02	−0.06
71	C	−0.05	−0.26	−0.13	−0.06	−0.02	−0.03	−0.10	−0.05	−0.10
72	C	0.15	−0.13	−0.15	−0.01	0.16	0.19	0.10	0.06	−0.02
80	C	−0.01	−0.35	−0.28	−0.16	−0.03	−0.03	−0.11	−0.12	−0.12
88	C	−0.17	−0.25	−0.27	−0.23	−0.14	−0.08	−0.03	−0.02	−0.08
94	C	−0.06	−0.05	0.03	0.09	0.06	0.08	0.05	0.12	0.03
97	C	−0.08	−0.13	−0.15	−0.10	−0.06	−0.03	−0.01	0.03	−0.03
106	C	−0.22	−0.32	−0.27	−0.17	−0.17	−0.17	−0.15	−0.02	0.00
120	C	0.06	0.20	0.13	0.09	0.14	0.39	0.46	0.44	0.15
125	C	−0.04	0.06	0.00	0.09	0.24	0.41	0.35	0.39	0.31
128	C	−0.04	0.03	0.03	0.09	0.18	0.39	0.48	0.55	0.36
131	C	−0.12	0.00	0.00	0.19	0.26	0.50	0.49	0.51	0.22
139	C	0.09	0.10	0.08	0.15	0.21	0.39	0.48	0.49	0.29

**Table A2:** Basic information about the sampling locations in the alpine (A) and continental (C) biogeographical regions.

ID Plot		Number of Sampled Trees	Longitude	Latitude	Altitude (m)	Slope Gradient (°)	Exposure	Stand Age	Average Diameter (cm)	AVERAGE Height (m)	Stand Structure	European Forest Type
20	A	5	14°55′	45°16′	1055	7	SW	69	28	21	Uneven-aged	Mountainous beech forest
21	A	19	14°52′	45°10′	885	10	NW	54	24	16	Uneven-aged	Mountainous beech forest
22	A	11	15°04′	45°26′	614	10	E	100	29	22	Uneven-aged	Mountainous beech forest
23	A	6	15°05′	45°18′	637	7	SE	101	28	24	Uneven-aged	Mountainous beech forest
24	A	24	15°05′	45°10′	623	30	SW	73	36	24	Uneven-aged	Beech forest
25	A	24	15°05′	45°01′	681	20	N	77	26	20	Uneven-aged	Beech forest
26	A	24	15°05′	44°52′	829	30	NE	93	29	21	Uneven-aged	Beech forest
31	A	23	15°14′	45°18′	433	10	SE	64	20	18	Even-aged	Beech forest
47	C	19	16°06′	45°44′	440	30	SW	30	21	17	Even-aged	Beech forest
71	C	24	16°06′	45°18′	205	45	N	77	26	18	Even-aged	Mesophytic deciduous forest
72	C	24	17°33′	45°09′	452	35	NE	59	23	23	Even-aged	Beech forest
125	C	15	17°45′	45°44′	185	12	NW	118	35	33	Even-aged	Beech forest
131	C	13	14°53′	45°27′	532	45	NW	85	27	23	Even-aged	Mesophytic deciduous forest
38	C	8	15°30′	45°44′	423	20	S	35	15	14	Even-aged	Beech forest
59	C	6	15°54′	45°53′	690	17	SW	50	28	22	Even-aged	Mesophytic deciduous forest
60	C	18	15°54′	45°35′	181	11	E	49	20	20	Even-aged	Mesophytic deciduous forest
69	C	9	16°07′	45°53′	177	0	SW	70	32	23	Even-aged	Mesophytic deciduous forest
80	C	17	16°18′	45°01′	244	20	W	70	24	22	Even-aged	Beech forest
88	C	11	16°31′	46°10′	475	30	N	96	43	30	Even-aged	Mesophytic deciduous forest
94	C	15	16°43′	46°10′	268	37	NE	101	37	25	Even-aged	Beech forest
97	C	13	16°44′	45°35′	349	65	W	81	26	22	Even-aged	Mesophytic deciduous forest
106	C	6	16°57′	45°53′	199	6	NW	98	33	29	Even-aged	Beech forest
120	C	21	17°21′	45°36′	403	22	NW	117	29	26	Even-aged	Mesophytic deciduous forest
128	C	18	17°33′	45°18′	358	3	E	98	31	29	Even-aged	Beech forest
139	C	16	18°10′	45°27′	152	9	NW	54	26	24	Even-aged	Mesophytic deciduous forest

## References

[1] Watson R.T., Zinyowera M.C., Moss R.H. (1998). The Regional Impacts of Climate Change: An Assessment of Vulnerability. A Special Report of the Intergovernmental Panel on Climate Change Working Group II.

[2] Tecshe M. (1989). In Die Fichte II/2–Krankheiten.Schaden.

[3] Saxe H. (1993). Triggering and predisposing factors in the “Red” decline syndrome of Norway spruce (*Picea abies*). Trees.

[4] Modrzynski J. (2003). Defoliation of older Norway spruce (*Picea abies*/L./Karst.) stands in the Polish Sudety and Carpathian mountains. For. Ecol. Manag..

[5] Stocker T.F., Qin D., Plattner G.-K., Tignor M., Allen S.K., Boschung J., Nauels A., Xia Y.V.B., Bex V., Midgley P.M., IPCC (2013). Summary for Policymakers. Climate Change 2013: The Physical Science Basis. Contribution of Working Group I to the Fifth Assessment Report of the Intergovernmental Panel on Climate Change.

[6] Branković Č., Patarčić M., Güttler I., Srnec L. (2012). Near-future climate change over Europe with focus on Croatia in an ensemble of regional climate model simulations. Clim. Res..

[7] Zierl B. (2004). A simulation study to analyse the relations between crown condition and drought in Switzerland. For. Ecol. Manag..

[8] EEA Water Scarcity and Drought Events in Europe during the Last Decade. https://www.eea.europa.eu/data-and-maps/figures/main-drought-events-in-europe.

[9] Spinoni J., Antofie T., Barbosa P., Bihari Z., Lakatos M., Szalai S., Szentimrey T., Vogt J. (2013). An overview of drought events in the Carpathian Region in 1961–2010. Adv. Sci. Res..

[10] Lionello P. (2012). The Climate of the Mediterranean Region.

[11] Lionello P., Malanotte-Rizzoli P., Boscolo R., Alpert P., Artale V., Li L., Luterbacher J., May W., Trigo R., Tsimplis M. (2006). The Mediterranean climate: An overview of the main characteristics and issues. Dev. Earth Environ. Sci..

[12] Ognjenović M., Seletković I., Marušić M., Jonard M., Rautio P., Timmermann V., Tadić M.P., Lanšćak M., Ugarković D., Potočić N. (2023). The Effect of Environmental Factors on the Nutrition of European Beech (*Fagus sylvatica* L.) Varies with Defoliation. Plants.

[13] Ognjenović M., Seletković I., Potočić N., Marušić M., Tadić M.P., Jonard M., Rautio P., Timmermann V., Lovreškov L., Ugarković D. (2022). Defoliation Change of European Beech (*Fagus sylvatica* L.) Depends on Previous Year Drought. Plants.

[14] IPCC (2014). Climate Change 2014: Impacts, Adaptation, and Vulnerability. Part A: Global and Sectoral Aspects. Contribution of Working Group II to the Fifth Assessment Report of the Intergovernmental Panel on Climate Change.

[15] Beniston M., Stephenson D.B., Christensen O.B., Ferro C.A.T., Frei C., Goyette S., Halsnaes K., Holt T., Jylhä K., Koffi B. (2007). Future extreme events in European climate: An exploration of regional climate model projections. Climatic Change.

[16] Lehtonen I., Ruosteenoja K., Jylhä K. (2014). Projected changes in European extreme precipitation indices on the basis of global and regional climate model ensembles. Int. J. Climatol..

[17] Prislan P., Čufar K., De Luis M., Gričar J. (2018). Precipitation is not limiting for xylem formation dynamics and vessel development in European beech from two temperate forest sites. Tree Physiol..

[18] Poljanec A., Ficko A., Boncina A. (2010). Spatiotemporal dynamic of European beech (*Fagus sylvatica* L.) in Slovenia, 1970–2005. For. Ecol. Manag..

[19] Fritts H.C. (1976). Tree Rings and Climate.

[20] Ognjenović M., Levanič T., Potočić N., Ugarković D., Indir K., Seletković I. (2020). Interrelations of various tree vitality indicators and their reaction to climatic conditions on a European beech (*Fagus sylvatica* L.) plot. Šumarski List.

[21] Dobbertin M., Neumann M., Schroeck H.-W. (2013). Tree Growth Measurements in Long-Term Forest Monitoring in Europe. Dev. Environ. Sci..

[22] Spiecker H., Mielikäinen K., Köhl M., Skovsgaard J.P. (1996). Growth Trends in European Forests: Studies from 12 Countries.

[23] Seidling W., Ziche D., Beck W. (2012). Climate responses and interrelations of stem increment and crown transparency in Norway spruce, Scots pine, and common beech. For. Ecol. Manag..

[24] Dobbertin M. (2005). Tree growth as indicator of tree vitality and of tree reaction to environmental stress: A review. Eur. J. For. Res..

[25] Gutiérrez E. (1988). Dendroecological study of *Fagus silvatica* L. in the Montseny mountains (Spain). Acta Oecologica.

[26] Jump A.S., Hunt J.M., Peñuelas J. (2006). Rapid climate change-related growth decline at the southern range edge of *Fagus sylvatica*. Glob. Change Biol..

[27] Etzold S., Waldner P., Thimonier A., Schmitt M., Dobbertin M. (2014). Tree growth in Swiss forests between 1995 and 2010 in relation to climate and stand conditions: Recent disturbances matter. For. Ecol. Manag..

[28] Zimmermann J., Hauck M., Dulamsuren C., Leuschner C. (2015). Climate warming-related growth decline affects *Fagus sylvatica*, but not other broad-leaved tree species in Central European mixed forests. Ecosystems.

[29] Dulamsuren C., Hauck M., Kopp G., Ruff M., Leuschner C. (2016). European beech responds to climate change with growth decline at lower, and growth increase at higher elevations in the center of its distribution range (SW Germany). Trees.

[30] Seletković Z., Tikvić I., Prpić B., Matić S. (2003). The Ecological Constitution of Common Beech. Common Beech in (Fagus sylvatica L.) in Croatia.

[31] Tinner W., Lotter A. (2006). Holocene expansion of *Fagus sylvatica* and *Abies alba* in Central Europe: Where are we after eight decades of debate?. Quat. Sci. Rev..

[32] Geßler A., Keitel C., Kreuzwieser J., Matyssek R., Seiler W., Rennenberg H. (2007). Potential risks for European beech (*Fagus sylvatica* L.) in a changing climate. Trees.

[33] Hacket-Pain A.J., Cavin L., Friend A.D., Jump A.S. (2016). Consistent limitation of growth by high temperature and low precipitation from range core to southern edge of European beech indicates widespread vulnerability to changing climate. Eur. J. For. Res..

[34] Di Filippo A., Biondi F., Čufar K., De Luis M., Grabner M., Maugeri M., Presutti Saba E., Schirone B., Piovesan G. (2007). Bioclimatology of beech (*Fagus sylvatica* L.) in the Eastern Alps: Spatial and altitudinal climatic signals identified through a tree-ring network. J. Biogeogr..

[35] Martinez del Castillo E., Longares L.A., Gričar J., Prislan P., Gil-Pelegrín E., Čufar K., de Luis M. (2016). Living on the Edge: Contrasted Wood-Formation Dynamics in *Fagus sylvatica* and Pinus sylvestris under Mediterranean Conditions. Front. Plant Sci..

[36] Čater M., Levanič T. (2019). Beech and silver fir’s response along the Balkan’s latitudinal gradient. Sci. Rep..

[37] Martinez del Castillo E., Zang C.S., Buras A., Hacket-Pain A., Esper J., Serrano-Notivoli R., Hartl C., Weigel R., Klesse S., Resco de Dios V. (2022). Climate-change-driven growth decline of European beech forests. Commun. Biol..

[38] Wigley T.M.L., Briffa K.R., Jones P.D. (1984). On the average value of correlated time series, with applications in dendroclimatology and hydrometeorology. J. Clim. Appl. Meteorol..

[39] Cvitan L., Hojsak T., Kozarić T., Likso T., Mikec K., Mikuš Jurković P., Mokorić M., Perčec Tadić M., Plačko-Vršnak D., Renko T. (2023). Climate Monitoring and Assessment for 2021.

[40] Bréda N., Badeau V. (2008). Forest tree responses to extreme drought and some biotic events: Towards a selection according to hazard tolerance?. Comptes Rendus Geosci..

[41] Primicia I., Camarero J.J., Janda P., Čada V., Morrissey R.C., Trotsiuk V., Bače R., Teodosiu M., Svoboda M. (2015). Age, competition, disturbance and elevation effects on tree and stand growth response of primary *Picea abies* forest to climate. For. Ecol. Manag..

[42] Rohner B., Kumar S., Liechti K., Gessler A., Ferretti M. (2021). Tree vitality indicators revealed a rapid response of beech forests to the 2018 drought. Ecol. Indic..

[43] Czúcz B., Gálhidy L., Mátyás C. (2011). Present and forecasted xeric climatic limits of beech and sessile oak distribution at low altitudes in Central Europe. Ann. For. Sci..

[44] Piovesan G., Biondi F., Bernabei M., Di Filippo A., Schirone B. (2005). Spatial and altitudinal bioclimatic zones of the Italian peninsula identified from a beech (*Fagus sylvatica* L.) tree-ring network. Acta Oecologica.

[45] Piovesan G., Biondi F., Filippo A.D., Alessandrini A., Maugeri M. (2008). Drought-driven growth reduction in old beech (*Fagus sylvatica* L.) forests of the central Apennines, Italy. Glob. Change Biol..

[46] Brang P.E. (1998). Sanasilva-Bericht 1997. Zustand und Gefahrdung des Schweizer Waldes—eine Zwischenbilanz nach 15 Jahren Waldschadenforschung.

[47] Cherubini P., Battipaglia G., Innes J.L. (2021). Tree Vitality and Forest Health: Can Tree-Ring Stable Isotopes Be Used as Indicators?. Curr. For. Rep..

[48] Cindrić K., Pasarić Z., Gajić-Čapka M. (2010). Spatial and temporal analysis of dry spells in Croatia. Theor. Appl. Climatol..

[49] Pilaš I., Medved I., Medak J., Perčec Tadić M., Medak D. (2016). Ecological, Typological Properties and Photosynthetic Activity (FAPAR) of Common Beech (*Fagus sylvatica* L.) Ecosystems in Croatia. SEEFOR–South-East Eropean For..

[50] Kramer K., Degen B., Buschbom J., Hickler T., Thuiller W., Sykes M.T., de Winter W. (2010). Modelling exploration of the future of European beech (*Fagus sylvatica* L.) under climate change—Range, abundance, genetic diversity and adaptive response. For. Ecol. Manag..

[51] Mihajlović D. (2006). Monitoring the 2003–2004 meteorological drought over Pannonian part of Croatia. Int. J. Climatol..

[52] Bréda N., Huc R., Granier A., Dreyer E. (2006). Temperate forest trees and stands under severe drought: A review of ecophysiological responses, adaptation processes and long-term consequences. Ann. For. Sci..

[53] Gajić-Čapka M., Perčec Tadić M., Patarčić M. (2003). Digitalna godišnja oborinska karta Hrvatske. Hrvat. Meteorološki Čas..

[54] Zaninović K., Srnec L., Perčec Tadić M. (2004). Digitalna godišnja temperaturna karta Hrvatske. Hrvat. Meteorološki Čas..

[55] Tikvić I., Seletković Z., Ugarković D., Posavec S., Španjol Ž. (2008). Dieback of Silver Fir (*Abies alba* Mill.) on Northern Velebit (Croatia). Period. Biol..

[56] Kolář T., Čermák P., Trnka M., Žid T., Rybníček M. (2017). Temporal changes in the climate sensitivity of Norway spruce and European beech along an elevation gradient in Central Europe. Agric. For. Meteorol..

[57] Eichhorn J., Roskams P., Potočić N., Timmermann V., Ferretti M., Mues V., Szepesi A., Durrant D., Seletković I., Schroeck H.-W. (2016). Part IV: Visual Assessment of Crown Condition and Damaging Agents. Manual on Methods and Criteria for Harmonized Sampling, Assessment, Monitoring and Analysis of the Effects of Air Pollution on Forests.

[58] Stokes M.A., Smiley T.L. (1968). An Introduction to Tree-Ring Dating.

[59] Levanič T. (2007). ATRICS–A new system for image acquisition in dendrochronology. Tree-Ring Res..

[60] Cook E.R., Peters K. (1981). The smoothing spline: A new approach to standardizing forest interior tree- ring width series for dendroclimatic studies. Tree-Ring Bull..

[61] Cook E.R. (1985). Time Series Analysis Approach to Tree Ring Standardization. Ph.D. Thesis.

[62] Bunn A.G. (2008). A dendrochronology program library in R (dplR). Dendrochronologia.

[63] (2019). R-Core-Team R: A Language and Environment for Statistical Computing.

[64] Cook E.R., Peters K. (1997). Calculating unbiased tree-ring indices for the study of climatic and environmental change. Holocene.

[65] Cook E.R., Holmes R.L. (1999). Program ARSTAN—Chronology Development with Statistical Analysis (User’s Manual for Program ARSTAN).

[66] Trouet V., Van Oldenborgh G.J. (2013). KNMI Climate Explorer: A web-based research tool for high-resolution paleoclimatology. Tree-Ring Res..

[67] Van Oldenborgh G.J. (1999). KNMI Climate Explorer.

[68] Hofstra N., Haylock M., New M., Jones P.D. (2009). Testing E-OBS European high-resolution gridded data set of daily precipitation and surface temperature. J. Geophys. Res..

[69] Beguería S., Vicente-Serrano S.M., Reig F., Latorre B. (2013). Standardized precipitation evapotranspiration index (SPEI) revisited: Parameter fitting, evapotranspiration models, tools, datasets and drought monitoring. Int. J. Climatol..

[70] Vicente-Serrano S.M., Beguería S., López-Moreno J.I. (2010). A multiscalar drought index sensitive to global warming: The Standardized Precipitation Evapotranspiration Index. J. Clim..

[71] Penzar B., Makjanić B. (1980). Osnovna Statistička Obrada Podataka u Klimatologiji.

[72] Zang C., Biondi F. (2015). treeclim: An R package for the numerical calibration of proxy-climate relationships. Ecography.

[73] Schweingruber F.H., Eckstein D., Serre Bachet F., Braker O.U. (1990). Identification, presentation and interpretation of event years and pointer years in dendrochronology. Dendrochronologia.

[74] Walther H. (1955). Die Klimadiagramme als Mittel zur Beurteilung der Klimaverhältnisse für ökologische, vegetationskundliche und landwirtschaftliche Zwecke. Ber. Der Dtsch. Bot. Ges..

